# Brain-wide TVA compensation allows rabies virus to retrograde target cell-type-specific projection neurons

**DOI:** 10.1186/s13041-022-00898-8

**Published:** 2022-01-29

**Authors:** Zengpeng Han, Nengsong Luo, Jiaxin Kou, Lei Li, Zihong Xu, Siyuan Wei, Yang Wu, Jie Wang, Chaohui Ye, Kunzhang Lin, Fuqiang Xu

**Affiliations:** 1 Key Laboratory of Magnetic Resonance in Biological Systems, State Key Laboratory of Magnetic Resonance and Atomic and Molecular Physics, National Center for Magnetic Resonance in Wuhan, Wuhan Institute of Physics and Mathematics, Innovation Academy for Precision Measurement Science and Technology, Chinese Academy of Sciences-Wuhan National Laboratory for Optoelectronics, Wuhan, 430071 People’s Republic of China; 2 University of Chinese Academy of Sciences, Beijing, 100049 People’s Republic of China; 3 The Brain Cognition and Brain Disease Institute (BCBDI), Shenzhen Key Laboratory of Viral Vectors for Biomedicine, Shenzhen Institute of Advanced Technology, Chinese Academy of Sciences, Shenzhen, 518055 People’s Republic of China; 4 Shenzhen-Hong Kong Institute of Brain Science-Shenzhen Fundamental Research Institutions, NMPA Key Laboratory for Research and Evaluation of Viral Vector Technology in Cell and Gene Therapy Medicinal Products, Shenzhen, Key Laboratory of Quality Control Technology for Virus-Based Therapeutics, Guangdong Provincial Medical Products Administration, Shenzhen, 518055 People’s Republic of China; 5 Wuhan National Laboratory for Optoelectronics, Huazhong University of Science and Technology, Wuhan, 430074 People’s Republic of China; 6 Department of Pathophysiology, Key Lab of Neurological Disorder of Education Ministry, School of Basic Medicine, Tongji Medical College, Huazhong University of Science and Technology, Wuhan, People’s Republic of China; 7 College of Life Sciences, Wuhan University, Wuhan, People’s Republic of China; 8 HongYi Honor College, Wuhan University, Wuhan, People’s Republic of China; 9 Shenzhen-Hong Kong Institute of Brain Science-Shenzhen Fundamental Research Institutions, Shenzhen, 518055 People’s Republic of China; 10 Center for Excellence in Brain Science and Intelligence Technology, Chinese Academy of Sciences, Shanghai, 200031 People’s Republic of China

**Keywords:** Retrograde tracing, Rabies virus, AAV-PHP.eB, Cell-type-specific neurons, Projection neurons

## Abstract

Retrograde tracers based on viral vectors are powerful tools for the imaging and manipulation of upstream neural networks projecting to a specific brain region, and they play important roles in structural and functional studies of neural circuits. However, currently reported retrograde viral tracers have many limitations, such as brain area selectivity or the inability to retrograde label genetically defined brain-wide projection neurons. To overcome these limitations, a new retrograde tracing method, AAV-PHP.eB assisted retrograde tracing systems (PARTS) based on rabies virus, was established through brain-wide TVA-dependent targeting using an AAV-PHP.eB that efficiently crosses the blood–brain barrier in C57BL/6 J mice, and complementation of EnvA-pseudotyped defective rabies virus that specifically recognizes the TVA receptor. Furthermore, combined with Cre transgenic mice, cell-type-specific PARTS (cPARTS) was developed, which can retrograde label genetically defined brain-wide projection neurons. Our research provides new tools and technical support for the analysis of neural circuits.

## Introduction

The neural network, which is formed by a large number of neurons of different types connected through synapses, is the structural basis of the brain’s complex activities. Understanding the connections between neurons in the nervous system is the challenge of current neuroscience research. Retrograde viral tracers can be used to map and manipulate the upstream neural network projecting to specific brain regions, making them the most promising potential tools for dissecting the structural and functional connections of neural circuits [1, 2]. Currently, the most commonly used retrograde viral tracers are engineered rabies virus (RV) and adeno-associated virus (AAV), such as N2cG-pseudotyped defective rabies virus (N2cG-RV-ΔG) [3], AAV2-retro [1] and AAV9-retro [4]. Among them, RV, as one of the earliest reported viral tracers due to its unique properties of axon terminal absorption and rapid gene expression, has been used for targeting long-projecting neuronal circuit assemblies [5–8]. AAV2-retro can be used not only to express fluorescent probes to analyse the structural connections of neural networks but also to express functional probes to monitor and manipulate neuronal activities [1]. However, AAV2-retro has brain region selectivity, mainly infecting cortical neurons, and N2cG-RV-ΔG can only broad-spectrum retrograde infect projection networks that are not cell-type-specific [3]. Therefore, there is an urgent need to establish new retrograde labelling methods for the analysis of genetically defined brain-wide projection neurons received by specific brain regions.

RV-ΔG pseudotyped with the avian sarcoma leucosis virus glycoprotein EnvA can specifically recognize and infect neurons that express the TVA receptor, an avian receptor protein that is absent in mammalian cells [9]. TVA can be expressed by transgenic animals or viral vectors, and retrograde infection of the EnvA-pseudotyped virus along the axon terminal can be restricted to directly input neurons [10, 11], but the preparation cycle of transgenic animals is long, and the cost is high. By combining transgenic animals and Cre-dependent viral vectors that express TVA, mapping of cell type-specific projection neurons can be achieved. However, there has not yet been a whole-brain tracing technology based on this system.

As a helper virus, the infectious properties of AAV depend on its capsid protein. AAV9 is widely used for neural circuit labelling because of its good effect on central nervous system infection among all AAV serotypes and has shown the ability to pass through the blood–brain barrier (BBB) after peripheral vascular administration [12–14]. Deverman et al. [15] conceived a Cre recombination-based AAV directed evolution strategy to isolate a novel engineered AAV9 capsid, named PHP.B, with the insertion of a 7-amino-acid sequence TLAVPFK into the VP1 capsid protein, which was shown to outperform the standard AAV9 in transducing neurons after intravenous injection. Moreover, an enhanced version named PHP.eB is more efficient in crossing the blood–brain barrier in C57BL/6 J mice and lowers the viral load required to transduce the majority of CNS neurons [16].

Through AAV vectors that can penetrate the blood–brain barrier, whole-brain delivery of exogenous proteins can be achieved; thus, we designed AAV-PHP.eB-assisted retrograde tracing systems (PARTS) based on RV. Through the transduction of the TVA receptor using AAV-PHP.eB to the whole brain in C57BL/6 J mice, EnvA-pseudotyped defective rabies virus could label brain-wide upstream neurons of a defined brain area. Furthermore, combined with Cre transgenic mice, cell-type-specific PARTS (cPARTS) was developed, which can retrograde label genetically defined brain-wide projection neurons.

The ventral tegmental area (VTA), a midbrain structure with multiple cell types, is known to integrate aversive and rewarding stimuli [17–21]. The VTA contains three types of neurons. The most abundant are dopaminergic neurons, which make up 60–65% of the total neurons, followed by GABAergic neurons, which make up approximately 30–35% of total neurons, and a small number of glutamatergic neurons, comprising approximately 2–3% of the total [22]. VTA GABA neurons have diverse functions, influencing dopaminergic activity through local inhibitory control and exerting dopamine (DA)-independent effects through projections to distal brain regions [23]. As a proof of principle, we used PARTS and cPARTS to trace the upstream input of the VTA area. Our research provides new tools and technical support for the analysis of neural circuits.

## Results

### Establishment of a new retrograde tracing method

Receptor compensation can mediate more effective retrograde labelling of the virus. For example, canine adenovirus type-2 (CAV-2) has enhanced retrograde labelling efficiency by compensating the coxsackievirus and adenovirus receptor (CAR) [24]. After compensation of the AAV receptor (AAVR) in input neurons, AAV2 can transport the upstream connection that the previously reported AAV can hardly label [25]; TVA can mediate effective retrograde infection of EnvA envelope pseudotyped virus [26]. However, whether effective and broad-spectrum retrograde labelling can be achieved by whole-brain receptor compensation is unknown. To verify this strategy, we established a PARTS strategy, including a TVA-expressing AAV-PHP.eB, which can efficiently transduce brain neurons across the blood–brain barrier, and an EnvA envelope pseudotyped defective rabies virus that can specifically recognize and infect TVA-positive neurons. Furthermore, we developed cell-type-specific PARTS (cPARTS), in which TVA expression is Cre-dependent.

**Fig. 1:**
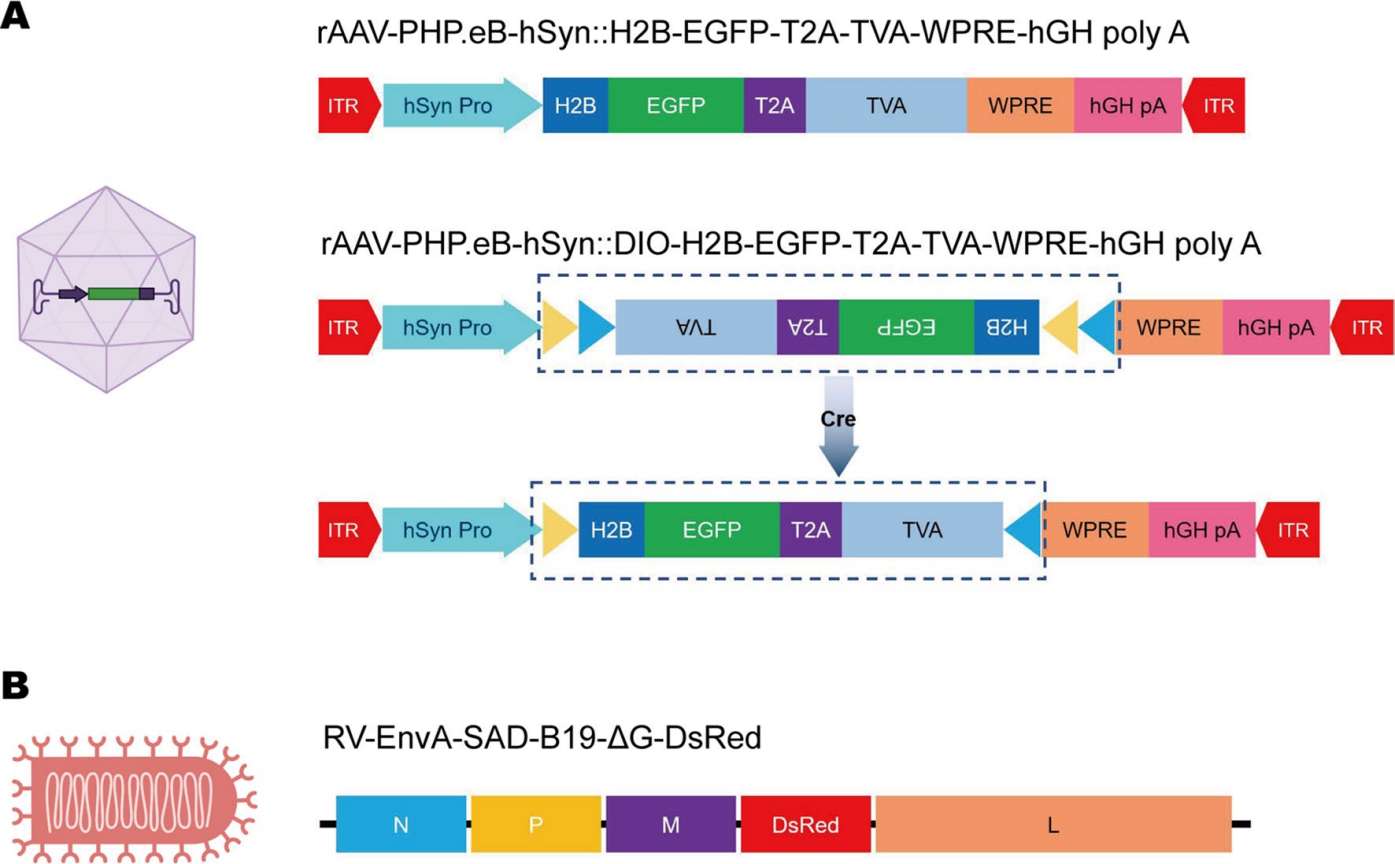
Schematic diagram of viral vectors carrying functional elements in PARTS and cPARTS strategies. **A** TVA-expressing vectors that can efficiently transduce brain neurons across the blood–brain barrier. The expression is direct and Cre-dependent. The AAV serotype used here is PHP.eB. **B** EnvA-pseudotyped glycoprotein (G)-defective rabies virus that can specifically recognize and infect TVA-positive neurons

Based on this design, Fig. 1 illustrates the functional elements carried in the viral vector in PARTS. We used AAV-PHP.eB carrying TVA fused with nuclear EGFP, and in cPARTS, TVA expression was Cre-dependent (Fig. 1A). The EnvA envelope pseudotyped SAD-B19-ΔG-DsRed used in PARTS and cPRATS is shown in Fig. 1B.

### A new retrograde tracing method effectively labels upstream neurons

**Fig. 2:**
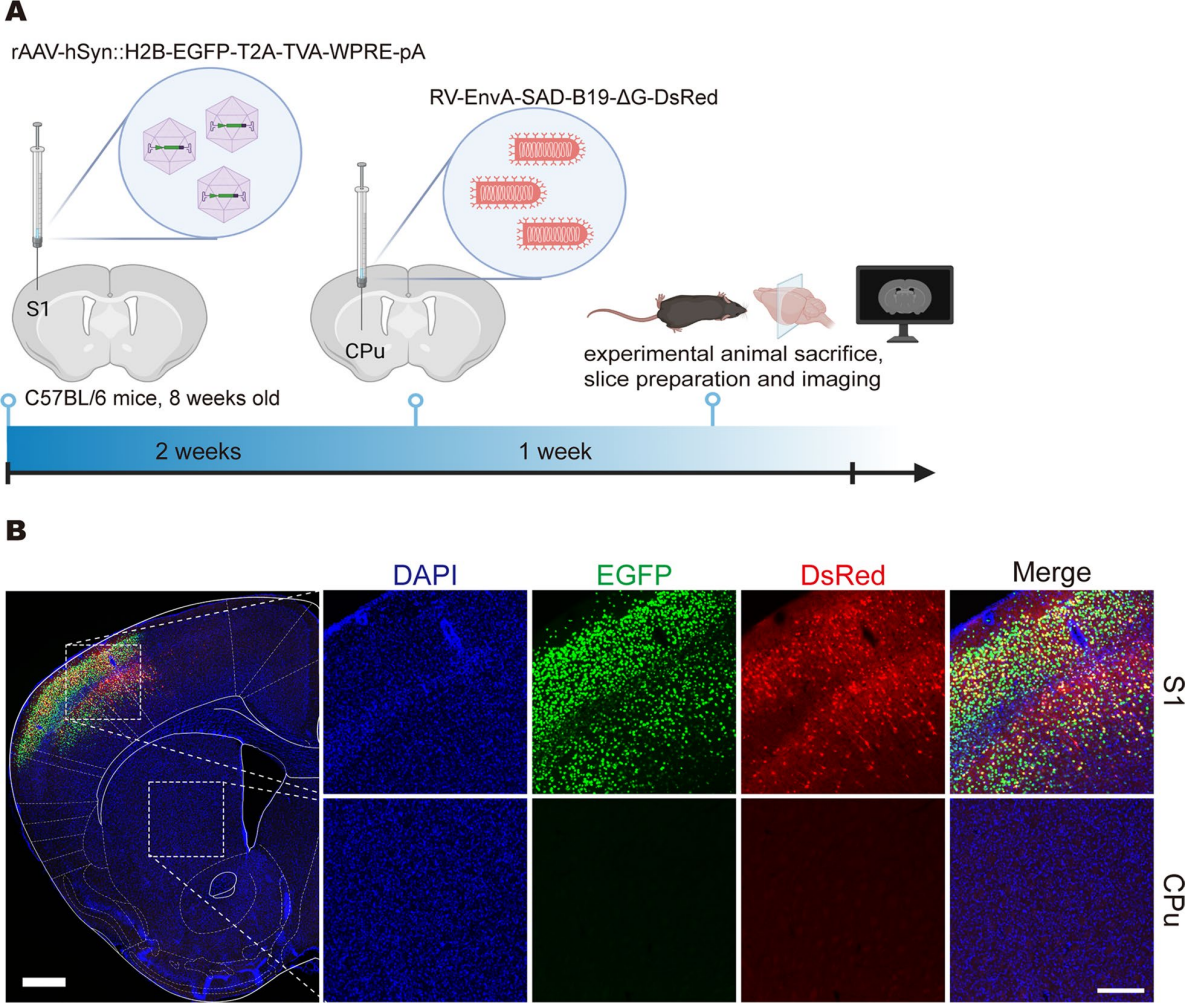
Local injection to verify the retrograde labelling of the PARTS method. **A** rAAV-hSyn::H2B-EGFP-T2A-TVA was injected into the S1 of C57BL/6 J mice. At 2 weeks postinjection, RV-EnvA-SAD-B19-ΔG-DsRed was injected into the CPu, and mice were sacrificed after another week. **B** Fluorescence expression at the injection site (CPu) and the upstream brain region (S1). In the large picture on the left, scale bar = 500 μm; in the small pictures on the right, scale bar = 200 μm

**Fig. 3:**
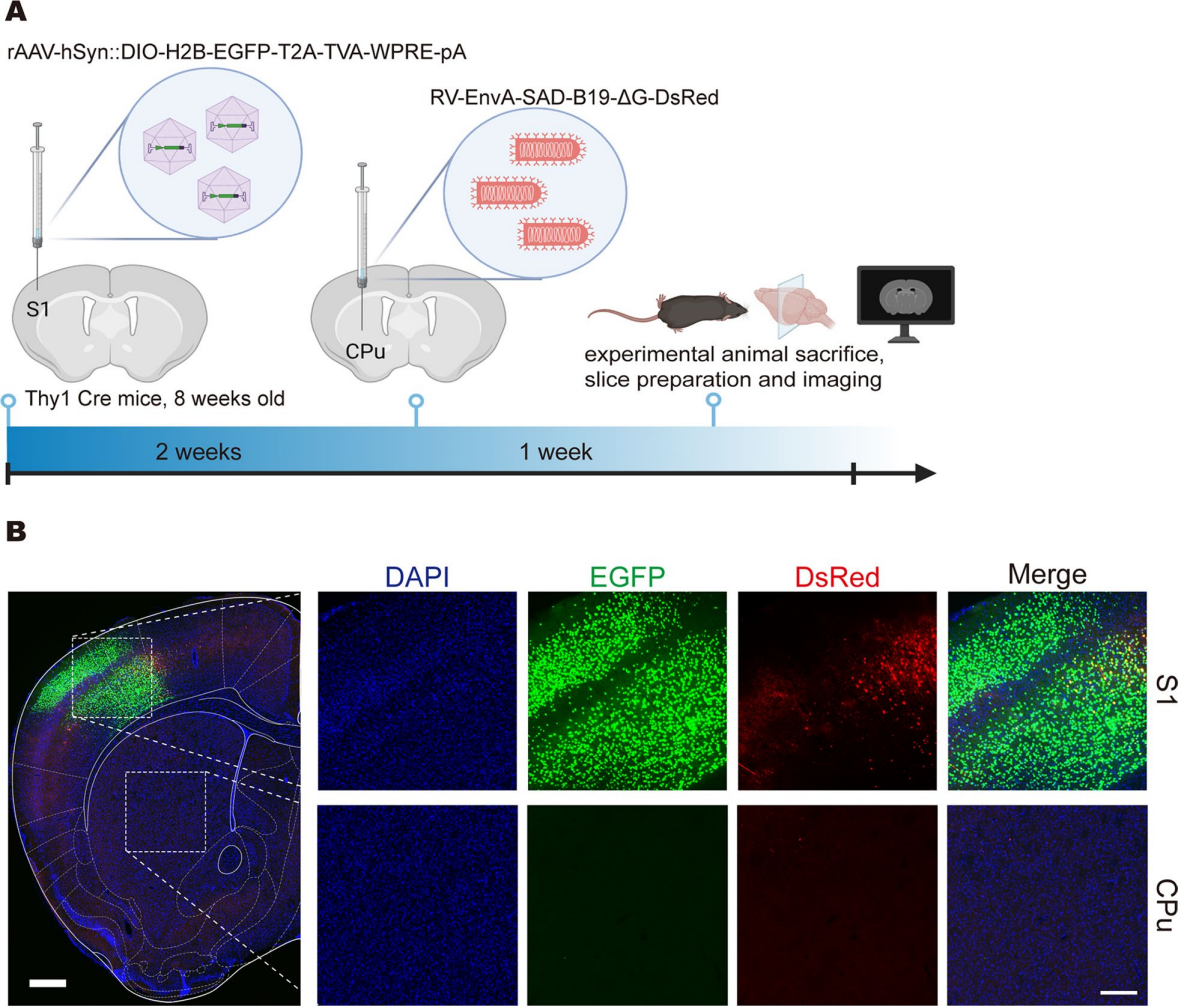
Local injection to verify the retrograde labelling of the cPARTS method. **A** rAAV-hSyn::DIO-H2B-EGFP-T2A-TVA was injected into the S1 of Thy1-Cre mice, RV-EnvA-SAD-B19-ΔG-DsRed was injected into the CPu after 2 weeks, and then mice were sacrificed in another week. **B** Fluorescence expression at the injection site (CPu) and the upstream brain region (S1). In the large picture on the left, scale bar = 500 μm; in the small pictures on the right, scale bar = 200 μm

AAV-PHP.eB can be used not only for whole brain infection by tail vein injection but also for in situ infection through local injection. Therefore, AAV-PHP.eB can also be used as a helper virus to analyse the connection between the two brain regions. To verify whether the helper virus can effectively mediate the retrograde labelling of EnvA-pseudotyped rabies virus, we tested PARTS (Fig. 2) and cPARTS (Fig. 3) in the S1 → CPu circuit. rAAV-PHP.eB-hSyn::H2B-EGFP-T2A-TVA and rAAV-PHP.eB-hSyn::DIO-H2B-EGFP-T2A-TVA were injected into the S1 of C57BL/6 J mice and Thy1-Cre transgenic mice, respectively, and RV-EnvA-SAD-B19-ΔG-DsRed was injected into the CPu after 2 weeks. The mice were sacrificed after another week, and the cortical region projecting to the CPu was imaged by an Olympus VS120 Slide Scanner microscope (Figs. 2A, 3A). Both green and red fluorescent signals appeared in S1, and red fluorescence was colabelled with green fluorescence (Figs. 2B, 3B). Moreover, no DsRed fluorescent signals of RV-EnvA were observed in the injection site CPu. These results showed that PARTS and cPARTS could retrogradely label projection neurons after injection into specific brain areas.

### Use of the new retrograde tracing method to trace the broad-spectrum upstream input network of the VTA

**Fig. 4:**
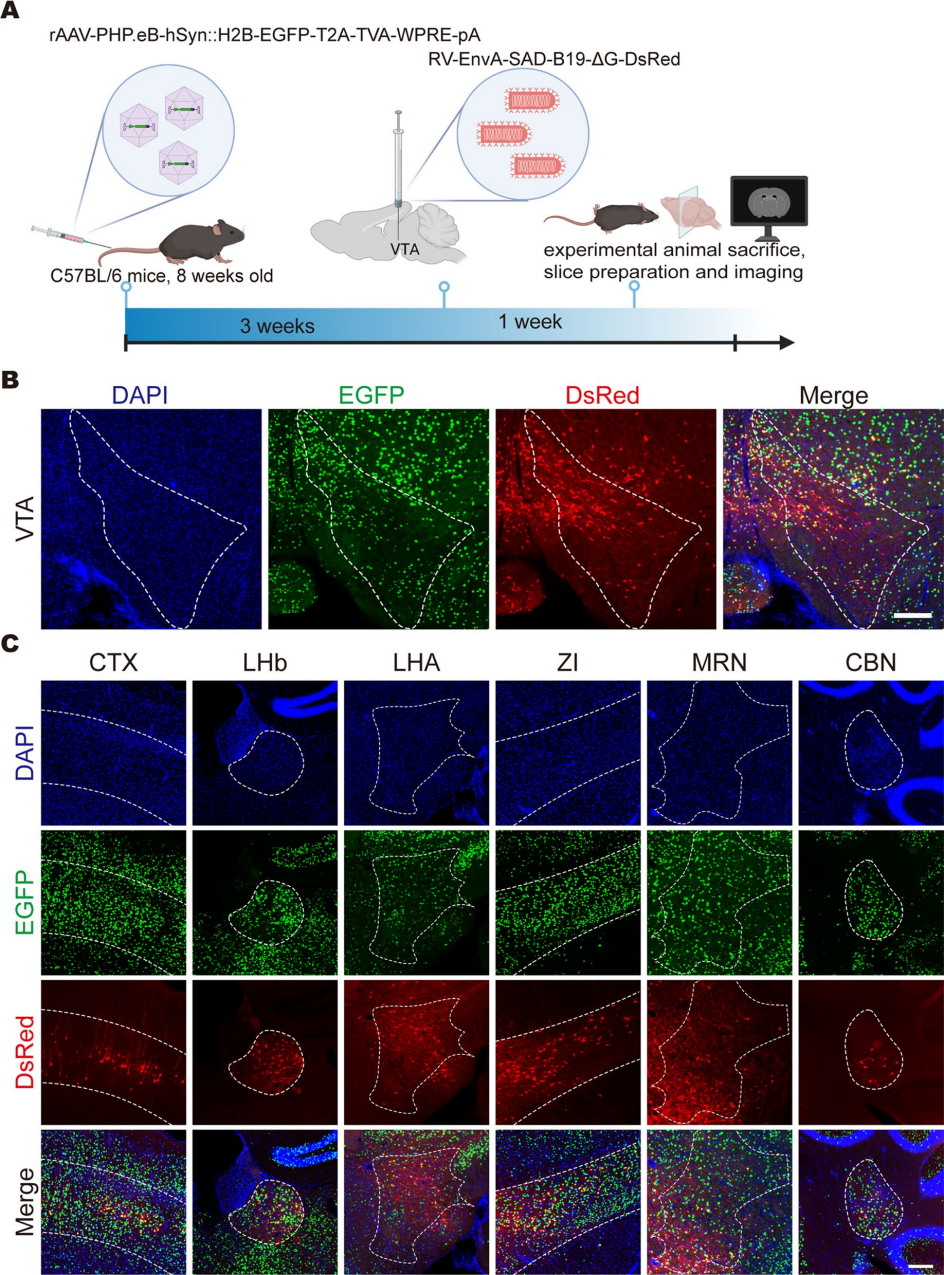
Use of the PARTS method to trace the broad-spectrum upstream input network of the VTA. **A** Schematic diagram of retrograde tracing by the PARTS method. Helper virus that expresses the TVA receptor fused with nuclear EGFP was intravenously injected into C57BL/6 J adult mice, and RV-EnvA-SAD-B19-ΔG-DsRed was injected into the VTA after 3 weeks. Then, the mice were sacrificed after another week, and the upstream inputs of the VTA were imaged. **B** Fluorescence expression at the injection site (VTA), scale bar = 200 μm. **C** Fluorescence expression in some upstream brain regions of the VTA. Red fluorescence signals were colabelled with green fluorescence signals, scale bar = 200 μm. *CTX* cerebral cortex, *LHb* lateral habenula, *LHA* lateral hypothalamic area, *ZI* zona incerta, *MRN* midbrain reticular nucleus, *CBN* cerebellar nuclei

To verify whether PARTS can be used for brain-wide retrograde tracing, rAAV-PHP.eB-hSyn-H2B-EGFP-T2A-TVA was intravenously injected into C57BL/6 J adult mice. At 3 weeks postinjection, RV-EnvA-SAD-B19-ΔG-DsRed was injected into the VTA, the mice were sacrificed after another week, and the upstream inputs of the VTA were imaged (Fig. 4A). We found that RV-EnvA-SAD-B19-ΔG-DsRed could infect the injection site (Fig. 4B) and upstream brain regions (Fig. 4C), including the cerebral cortex (CTX), lateral habenula (LHb), lateral hypothalamic area (LHA), zona incerta (ZI), midbrain reticular nucleus (MRN), and cerebellar nuclei (CBN), among others. More than 96% of rabies-labelled cells are also positive for AAV-TVA tags (EGFP), and several cells have only red signals because the weak expression of the TVA receptor can result in RV-EnvA infection; in this case, EGFP signals are not significant [10]. These results indicate that PARTS can be used to retrograde trace brain-wide input neurons of the specific brain regions of our choice.

### Use of the new retrograde tracing method to label the cell-type-specific input network upstream of the VTA

**Fig. 5:**
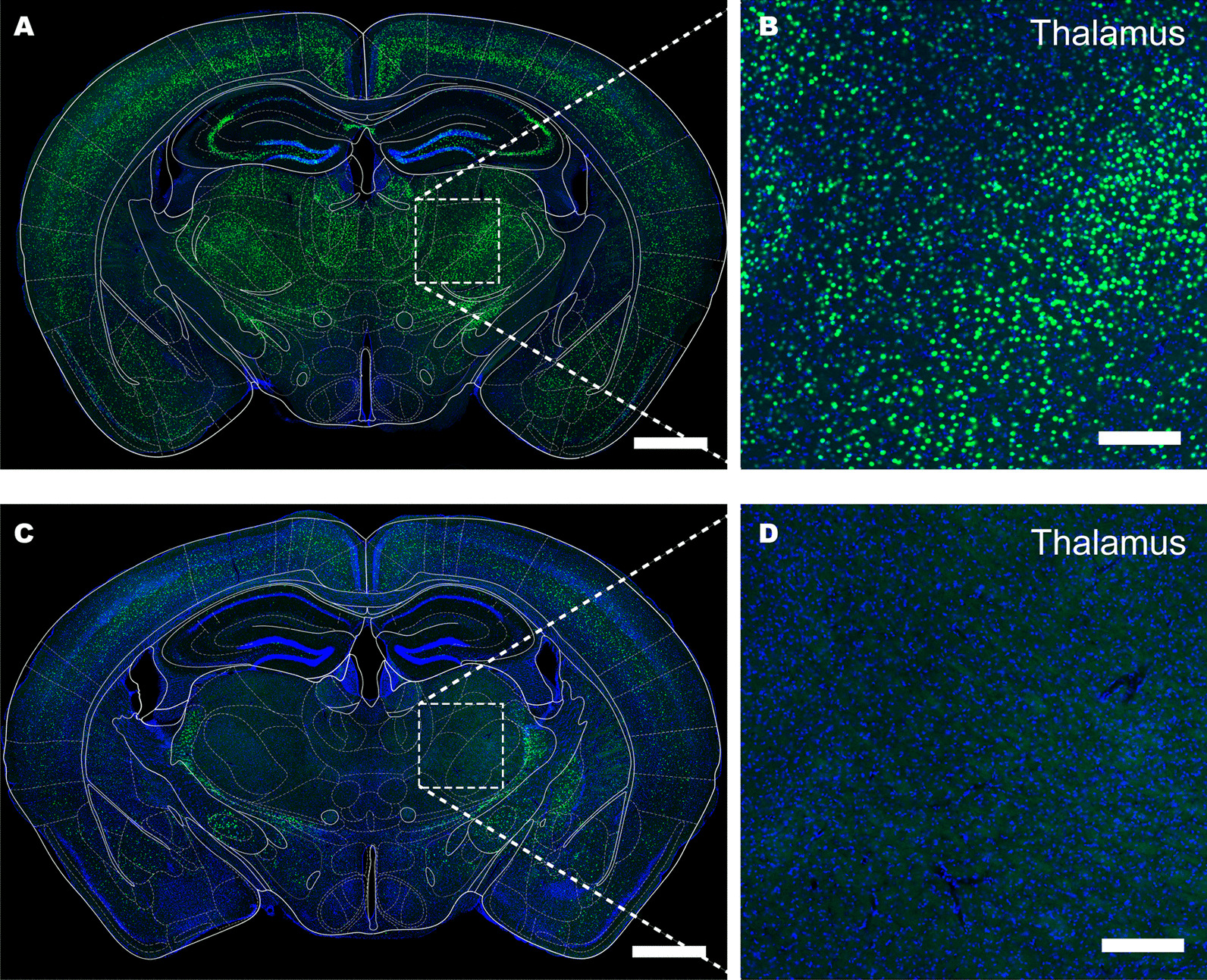
Distribution of GABAergic neurons in the whole brain. **A** Labelling of rAAV-PHP.eB-hSyn-H2B-EGFP-T2A-TVA in C57BL/6 J mice, scale bar = 1 mm. **B** Green fluorescence signals in the thalamus of C57BL/6 J mice, scale bar = 200 μm. **C** Conditional labelling of rAAV-PHP.eB-hSyn-DIO-H2B-EGFP-T2A-TVA in VGAT-Cre transgenic mice, scale bar = 1 mm. (D) Green fluorescence signals in the thalamus of VGAT-Cre mice, scale bar = 200 μm

**Fig. 6:**
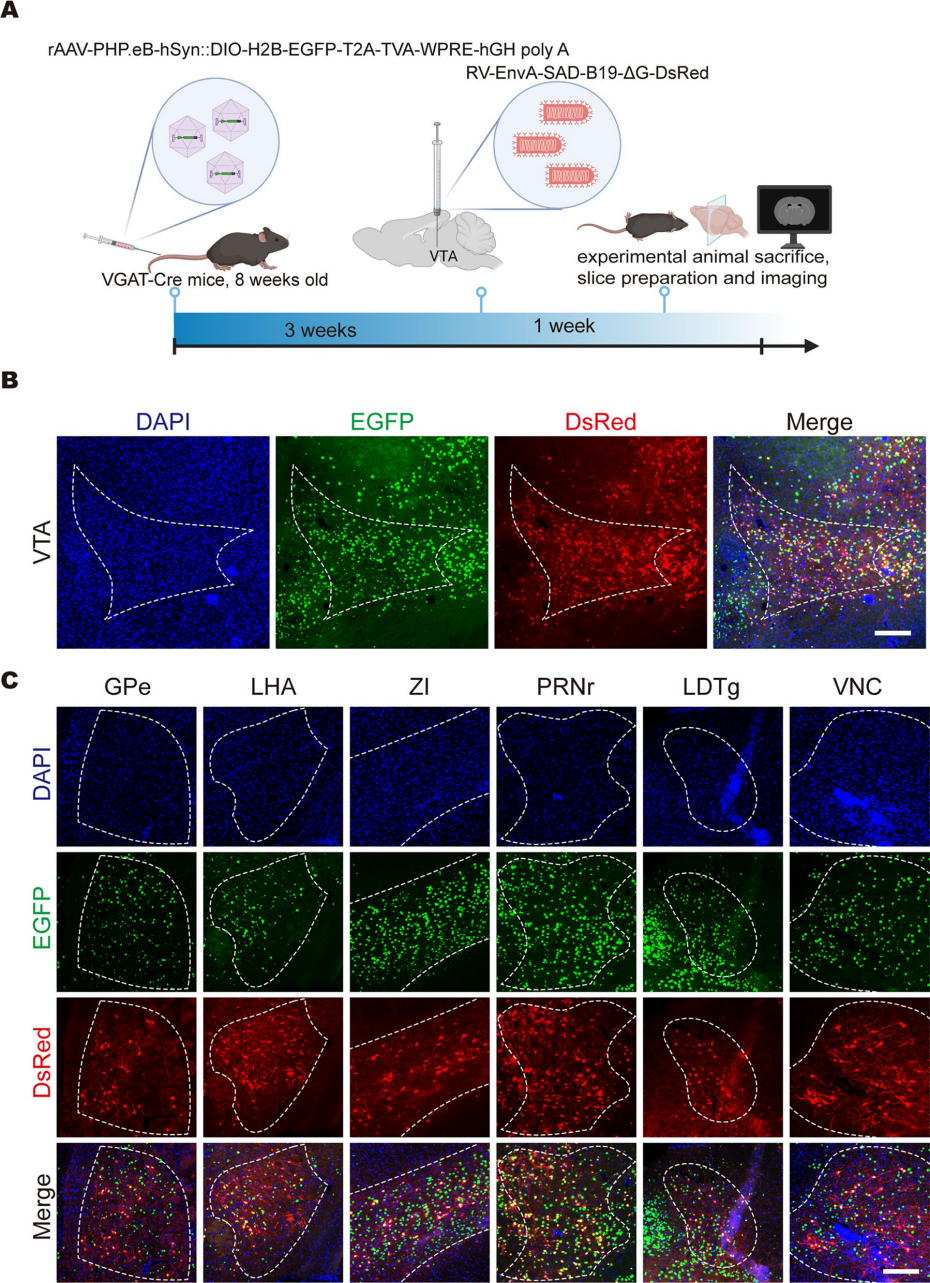
Use of the cPARTS method to trace the cell-type-specific upstream input network of the VTA. **A** Schematic diagram of retrograde tracing by the cPARTS method. Cre-dependent helper virus that expresses the TVA receptor fused with nuclear EGFP was intravenously injected into VGAT-Cre adult mice, and RV-EnvA-SAD-B19-ΔG-DsRed was injected into the VTA after 3 weeks. Then, the mice were sacrificed after another week, and the upstream inputs of the VTA were imaged. **B** Fluorescence expression at the injection site (VTA), scale bar = 200 μm. **C** Fluorescence expression in some upstream brain regions of the VTA. Red fluorescence signals were colabelled with green fluorescence signals, scale bar = 200 μm. *GPe* globus pallidus, external segment; *LHA* lateral hypothalamic area; *ZI* zona incerta; *PRNr* pontine reticular nucleus; *LDTg* laterodorsal tegmental nucleus; *VNC* vestibular nuclei

Before using the cPARTS method to trace the cell-type-specific upstream input network of the VTA, rAAV-PHP.eB-hSyn-DIO-H2B-EGFP-T2A-TVA, which has Cre-dependent conditional EGFP expression, was intravenously injected into VGAT-Cre mice, and almost no green fluorescence signals of GABAergic neurons in the thalamus were labelled (Figs. 5C, D), consistent with the in situ hybridization (ISH) results published on the Allen Brain Atlas website. As a control, rAAV-PHP.eB-hSyn-H2B-EGFP-T2A-TVA was intravenously injected into C57BL/6 J mice, and obvious green fluorescence signals could be observed in the thalamus of C57BL/6 J mice (Fig. 5A, B). These results showed that rAAV-PHP.eB-hSyn-DIO-H2B-EGFP-T2A-TVA could specifically label GABAergic neurons in the whole brains of VGAT-Cre mice. After rAAV-PHP.eB-hSyn-H2B-EGFP-T2A-TVA was intravenously injected into adult C57BL/6 J mice for 3 weeks, RV-EnvA-SAD-B19-ΔG-DsRed was injected into the VTA, the mice were sacrificed after another week, and the upstream inputs of the VTA were imaged (Fig. 6A). We found that RV-EnvA-SAD-B19-ΔG-DsRed could infect the injection site (Fig. 6B) and upstream brain regions (Fig. 6C), including the globus pallidus, external segment (GPe), lateral hypothalamic area (LHA), zona incerta (ZI), pontine reticular nucleus (PRNr), laterodorsal tegmental nucleus (LDTg) and vestibular nuclei (VNC), among others. Moreover, fewer than five cells in a single brain area showed single-positive neurons of DsRed, which may be caused by low levels of TVA expression. These levels of leak might be undetectable by conventional methods such as fluorescent protein expression but can result in RV infection and high levels of viral gene expression [10, 27–29]. These results indicate that cPARTS can be used to retrograde trace brain-wide genetically defined input neurons of the specific brain regions of our choice.

### New strategies for retrograde tracing

**Fig. 7:**
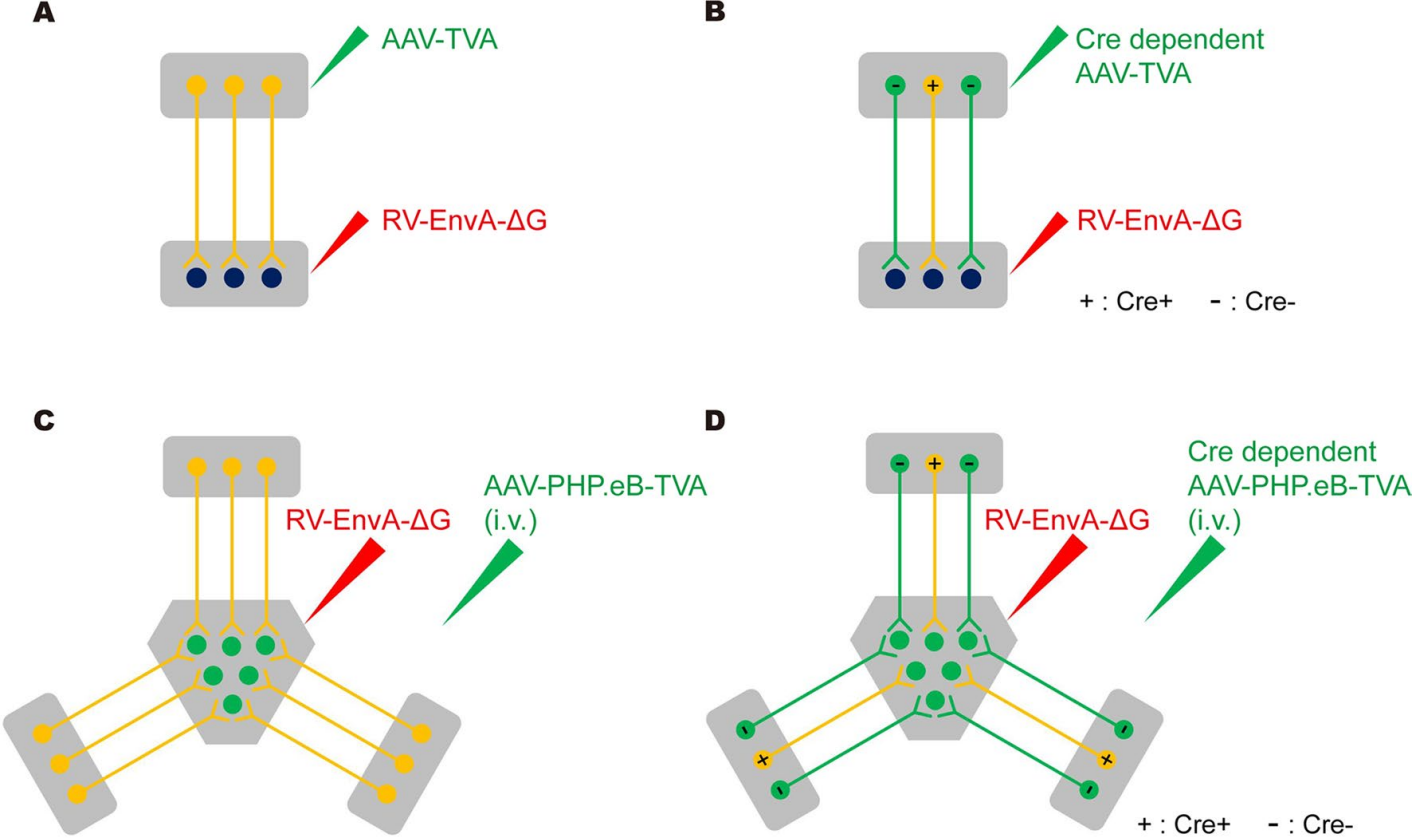
Summary of retrograde tracing strategies. **A** The PARTS strategy can realize traditional broad-spectrum retrograde tracing in two brain regions. **B** The cPARTS strategy can realize traditional cell type-specific retrograde tracing in two brain regions. **C** The PARTS strategy can realize broad-spectrum retrograde tracing in the whole brain. **D** The cPARTS strategy can realize cell type-specific retrograde tracing in the whole brain

Based on the above experimental results, we successfully established new retrograde tracing technologies, including PARTS and cPARTS. Their application strategies are summarized as follows: PARTS identifies neurons in the B region (local injection, Fig. 7A) or in brain-wide regions (intravenous injection, Fig. 7C) that project to a specific A region; cPARTS identifies cell-type-specific neurons in the B region (local injection, Fig. 7B) or in brain-wide regions (intravenous injection, Fig. 7D) that project to a specific A region.

## Discussion

Retrograde neurotropic viruses, which permit efficient gene transfer to the cell bodies of projection neurons from axonal terminals, have become essential tracers in the field of neuroscience by enabling the structural and functional analysis of genetically defined neural circuits. Due to their use of different receptors and infection mechanisms, the labelling characteristics of the various viruses are quite different [30, 31]. Such tropism is useful for the study of specific neural circuits, but in general, it lacks universality [3, 24]. In the present study, we developed PARTS and cPARTS strategies for the analysis of brain-wide projection neurons received by specific brain regions. AAV-PHP.eB, which can efficiently cross the blood–brain barrier in C57BL/6 J mice [16], is applied to the RV retrograde tracing system through whole-brain TVA compensation after intravenous injection, and broad-spectrum or cell-type-specific retrograde labelling in the whole brain can be achieved by EnvA-pseudotyped glycoprotein-deleted rabies virus. The retrograde tracing technique in two brain regions (Fig. 7A, B) can be widely used in the analysis of single neural circuits; moreover, our new methods can expand the labelling system to the brain-wide scale (Fig. 7C, D).

Some natural and engineered retrograde neurotropic viruses have been widely used in the study of neural circuits, such as rabies virus (RABV) [3, 7], lentivirus (LV) packaged by modified RVG [32–35], canine adenovirus (CAV) [36], herpes simplex virus (HSV) [37], and adeno-associated virus (AAV) [1, 38–40]. However, they have different labelling efficiencies and brain region selectivities [3, 26]. Modifying the surface structures of the viral envelope or capsid can improve tropism, but viruses still rely on cell surface molecules for uptake and transport and cannot gain entry into cells without the required receptors [24]. Receptor complementation approaches are a better strategy to overcome tropism and mediate more effective retrograde labelling of the virus. For example, canine adenovirus type-2 (CAV-2) has enhanced retrograde labelling efficiency by compensating for the coxsackievirus and adenovirus receptor (CAR) [24]. After compensation of the AAV receptor (AAVR) in input neurons, AAV2 can transport the upstream connection that the previously reported AAV can hardly label [25]; TVA can mediate effective retrograde infection of EnvA envelope pseudotyped virus [26]. Among them, TVA compensation for EnvA-pseudotyped virus permits retrograde labelling of cell-type-specific inputs because viruses pseudotyped with the avian sarcoma leucosis virus glycoprotein EnvA can infect only neurons that express exogenous TVA receptor, an avian receptor protein that is absent in mammalian cells [9]. Therefore, TVA receptor compensation for neurotropic viruses may be valuable tracers for targeting and manipulating broad-spectrum or genetically defined neural input networks.

We verified the whole-brain compensation effect of TVA carried by PHP.eB in C57BL/6 J and VGAT-Cre transgenic mice, and the distribution of GABAergic neurons in the whole brain was consistent with the in situ hybridization (ISH) results published on the Allen Brain Atlas website. We verified the PARTS and cPARTS labelling effects of brain-wide retrograde tracing in the VTA region, and significant fluorescence signals could be seen in several upstream regions (Figs. 4C, 6C). Among them, LHb is involved in reward, aversion, addiction and depression through descending interactions with several brain structures, including the ventral tegmental area (VTA) [41]. The LHA is implicated in feeding, food reward, and other motivated behaviours, and activation of all VTA-projecting LHA neurons enhances sucrose seeking [42]. de Git et al. [43] confirmed that activity of the ZI to VTA projection promotes feeding, which improves the understanding of the neurobiology of feeding behaviour and body weight regulation. Hernandez et al. [44] proved that GLP-1 receptor signalling in the LDTg attenuates cocaine seeking by activating GABAergic circuits that project to the VTA. Therefore, PARTS and cPARTS can be applied to analyse projection neurons related to important behaviours.

The VTA, which is an important brain region and is involved in various behaviours, is reported to connect with most brain regions; however, the upstream inputs of distinct cell types have not yet been completely defined. Based on the PARTS and cPARTS strategies, we can provide more information and anatomical evidence to help understand the precise connections and diverse functions of specific brain regions. Among the commonly used retrograde tracing tools, AAV2-retro has difficulty achieving unbiased retrograde labelling and prefers to infect cortical neurons; N2cG-RV-ΔG can only broad-spectrum retrograde infect projection networks that are not cell type-specific [3]. The PARTS/cPARTS strategies overcome the shortcomings of the above two. On the basis of broad-spectrum labelling, cell type-specific retrograde tracing can be easily achieved using transgenic animals and conditionally expressed TVA elements carried by AAV-PHP.eB. In addition, many different viral tracers or methods for retrograde tracing have been used in the neuroscience field. Here, we provide a more detailed comparison (pros and cons) among them (Table 1), and researchers can choose the appropriate viral tracers according to their different experimental needs.

**Table 1:** Comparison of viral tracers and methods of retrograde tracing

Method/virus	Efficiency	Preparation	Toxicity	Brain region selectivity	Cell-type-specific
AAV2-retro	High	Easy	Low	Yes	Yes
AAV & AAVR	Medium	Easy	Low	No	Yes
CAV2	Low	Easy	Low	Yes	Yes
CAV2 & CAR	Medium	Easy	Low	No	Yes
RV-ΔG(N2cG)	High	Easy	High	No	No
PARTS	High	Easy	High	No	No
cPARTS	High	Easy	High	No	Yes

Efficiency and brain region selectivity of viral tracers are relative to other viruses, and in most cases, researchers can choose the appropriate viral tracers according to their different experimental needs

Whole-brain delivery of the TVA receptor depends on the AAV mutant PHP.eB, which efficiently crosses the blood–brain barrier, but the neurotropic properties of AAV-PHP.eB are limited to C57BL/6 J mice [45, 46]; therefore, expanding the scope of application of PARTS currently requires the acquisition of AAV mutants that efficiently cross the blood–brain barrier in a broader range of species. According to previous reports, intravenous administration of 1 × 10^11^ vg of AAV-PHP.eB transduced 69% of cortical and 55% of striatal neurons in adult mice [16]. Although the dosage was increased, intravenous delivery of AAV-PHP.eB could only transduce a fraction of whole neuron populations. This limitation may be compensated by the development of AAV with higher infection efficiency.

In summary, we have proven that brain-wide TVA compensation can mediate efficient retrograde targeting of the RV to projection neurons. Our research provides new tools and technical support for the analysis of neural circuits.

## Materials and methods

### Vector construction and virus preparation

To construct pAAV-PHP.eB-hSyn-DIO-H2B-EGFP-T2A-TVA-WPRE-pA, the H2B-EGFP-T2A-TVA sequence was synthesized and inserted into pAAV-hSyn-DIO-EGFP-WPRE-hGH polyA (BrainCase, ShenZhen) digested by the restriction enzymes NheI and AscI (New England Biolabs). To construct the pAAV-PHP.eB-hSyn-H2B-EGFP-T2A-TVA-WPRE-pA, the H2B-EGFP-T2A-TVA sequence was amplified and inserted into the pAAV-hSyn-DIO-EGFP-WPRE-hGH polyA digested by the restriction enzymes SalI and HindIII (New England Biolabs). AAV vectors, including rAAV-PHP.eB-hSyn-DIO-H2B-EGFP-T2A-TVA-WPRE-pA and rAAV-PHP.eB-hSyn-H2B-EGFP-T2A-TVA-WPRE-pA, were produced in HEK-293 T cells cotransfected with pUCmini-iCAP-PHP.eB (Addgene plasmid #103005) and pAdDeltaF6 (Addgene plasmid #112867) and then purified by iodixanol gradient ultracentrifugation [47]. The purified rAAVs were titered by qPCR using the iQ SYBR Green Supermix kit (Bio–Rad). The titers of rAAV-PHP.eB-hSyn-H2B-EGFP-T2A-TVA-WPRE-pA and rAAV-PHP.eB-hSyn-DIO-H2B-EGFP-T2A-TVA-WPRE-pA were 5.28 × 10^12^ VG/mL and 1.1 × 10^13^ VG/mL, respectively. RV-EnvA-SAD-B19-ΔG-DsRed was prepared according to a previously reported method [48], and the titer was 5 × 10^7^ IU/mL.

### Animals

Adult male (8–10 weeks old) C57BL/6 J mice (Hunan SJA Laboratory Animal Company) and VGAT-Cre transgenic mice (The Jackson Laboratory) were used for all experiments. The mice were housed in the appropriate environment with a 12/12-h light/dark cycle, and water and food were supplied ad libitum. All surgical and experimental procedures were performed in accordance with the guidelines formulated by the Animal Care and Use Committee of the Innovation Academy for Precision Measurement Science and Technology, Chinese Academy of Sciences.

### Virus injection

The stereotactic injection coordinates were selected according to Paxinos and Franklin’s *The Mouse Brain in Stereotaxic Coordinates*, 4th edition [49]. The stereotactic coordinates for S1 were as follows: AP: + 0.50 mm; ML: ± 3.00 mm; DV: − 2.00 mm from bregma. The CPu values were as follows: AP: + 0.75 mm; ML: ± 2.00 mm; and DV: − 3.30 mm from bregma. The VTA values were as follows: AP: − 3.20 mm; ML: ± 0.45 mm; DV: − 4.30 mm from bregma. Eight- to ten-week-old C57BL/6 J mice and VGAT-Cre transgenic mice (20–25 g) were used for virus injection, and the standard injection process was performed as previously reported [3]. After 2 weeks of AAV virus expression in S1, RV-EnvA-SAD-B19-ΔG-DsRed was injected into the CPu of C57BL/6 J mice, and the mice were sacrificed at 7 days postinjection using the conventional cardiac perfusion method. For brain-wide retrograde tracing, TVA-expressing rAAV-PHP.eB-hSyn-DIO-H2B-EGFP-T2A-TVA and rAAV-PHP.eB-hSyn-H2B-EGFP-T2A-TVA (4 × 10^11^ VG/mouse) were intravenously injected into VGAT-Cre transgenic adult mice and C57BL/6 J mice, respectively. After 3 weeks, RV-EnvA-SAD-B19-ΔG-DsRed was injected into the VTA, and the mice were sacrificed at 7 days postinjection.

### Slice preparation and imaging

Slice preparation and imaging were completed according to previously reported methods [4]. The brains were soaked with 4% paraformaldehyde solution overnight, dehydration was accomplished at 37 ℃ with 30% sucrose solution, the brain was sectioned at a thickness of 40 μm using a frozen section instrument, and the brain slices were retrieved at 200-μm intervals. The brain slices were washed 3 times with phosphate-buffered saline (PBS) for 10 min each time. After 4',6-diamidino-2-phenylindole (DAPI) staining for 10 min, the brain slices were applied neatly on microscope slides and washed with PBS 3 times, followed by sealing with 70% glycerol. Imaging was performed using an Olympus VS120 Slide Scanner microscope (Olympus, Japan).

## Data Availability

The datasets used or analysed during the current study are available from the corresponding author on reasonable request.

## Abbreviations

AAV: Adeno-associated virus
PARTS: AAV-PHP.eB assisted retrograde tracing systems
cPARTS: Cell-type-specific PARTS
VG: Viral genomes
IU: Infection units, DAPI: 4',6-diamidino-2-phenylindole
CPu: Caudate putamen (striatum)
CTX: Cerebral cortex
LHb: Lateral habenula
LHA: Lateral hypothalamic area
ZI: Zona incerta
MRN: Midbrain reticular nucleus
CBN: Cerebellar nuclei
VTA: Ventral tegmental area
GPe: Globus pallidus, external segment
PRNr: Pontine reticular nucleus
LDTg: Laterodorsal tegmental nucleus
VNC: Vestibular nuclei
TH: Thalamus
